# Bump-Fabrication Technologies for Micro-LED Display: A Review

**DOI:** 10.3390/ma18081783

**Published:** 2025-04-14

**Authors:** Xin Wu, Xueqi Zhu, Shuaishuai Wang, Xuehuang Tang, Taifu Lang, Victor Belyaev, Aslan Abduev, Alexander Kazak, Chang Lin, Qun Yan, Jie Sun

**Affiliations:** 1 College of Physics and Information Engineering, Fuzhou University, Fuzhou 350100, China; 231127106@fzu.edu.cn (X.W.); 221127131@fzu.edu.cn (X.Z.); sy479380@163.com (S.W.); 221127083@fzu.edu.cn (X.T.); 231110008@fzu.edu.cn (T.L.); linchang@fjoel.cn (C.L.); qunfyan@gmail.com (Q.Y.); 2 Fujian Science & Technology Innovation Laboratory for Optoelectronic Information of China, Fuzhou 350108, China; 3 Faculty of Physics and Mathematics, State University of Education, Very Voloshinoi Str. 24, 141014 Mytishchi, Russia; vic_belyaev@mail.ru (V.B.); a_abduev@mail.ru (A.A.); alexkazak86@gmail.com (A.K.); 4 Department of Microscience and Nanotechnology, Chalmers University of Technology, 41296 Gothenburg, Sweden

**Keywords:** Micro-LED, bump fabrication, evaporation, electroplating, electroless plating, ball mounting, dip soldering, photosensitive conductive polymers, high-density interconnects

## Abstract

Micro Light Emitting Diode (Micro-LED) technology, characterized by exceptional brightness, low power consumption, fast response, and long lifespan, holds significant potential for next-generation displays, yet its commercialization hinges on resolving challenges in high-density interconnect fabrication, particularly micrometer-scale bump formation. Traditional fabrication approaches such as evaporation enable precise bump control but face scalability and cost limitations, while electroplating offers lower costs and higher throughput but suffers from substrate conductivity requirements and uneven current density distributions that compromise bump-height uniformity. Emerging alternatives include electroless plating, which achieves uniform metal deposition on non-conductive substrates through autocatalytic reactions albeit with slower deposition rates; ball mounting and dip soldering, which streamline processes via automated solder jetting or alloy immersion but struggle with bump miniaturization and low yield; and photosensitive conductive polymers that simplify fabrication via photolithography-patterned composites but lack validated long-term stability. Persistent challenges in achieving micrometer-scale uniformity, thermomechanical stability, and environmental compatibility underscore the need for integrated hybrid processes, eco-friendly manufacturing protocols, and novel material innovations to enable ultra-high-resolution and flexible Micro-LED implementations. This review systematically compares conventional and emerging methodologies, identifies critical technological bottlenecks, and proposes strategic guidelines for industrial-scale production of high-density Micro-LED displays.

## 1. Introduction

Display technology has evolved through successive generations of advancements, driving transformative upgrades in visual devices. The advent of the cathode-ray tube (CRT)-based color television in the 1940s marked a seminal milestone, catalyzing subsequent innovations in display systems. Subsequently, liquid crystal display (LCD) and organic light-emitting diode (OLED) technologies have been developed successively [1,2,3,4]. This evolutionary trajectory underscores persistent breakthroughs in display technology, particularly in achieving enhanced brightness, reduced power consumption, and ultrahigh resolution [5,6]. In recent years, Micro-LED technology, characterized by light-emitting diodes (LEDs) with lateral dimensions below 50 μm, has emerged as a focal point of research. Compared to LCD and OLED, Micro-LED exhibits more outstanding characteristics: higher brightness, lower power consumption, ultra-high levels of resolution and color saturation, a response speed reaching the nanosecond level, and a significantly extended service life [7,8,9,10]. Recent research has demonstrated that plasmonic effects can effectively enhance the luminous efficiency of Micro-LEDs [11,12,13], thereby reducing the device power consumption while optimizing the optical performance. These advantages make Micro-LED displays a leading candidate technology for next-generation visual devices, and they can be widely applied in smartwatches, smartphones, televisions, laptops, augmented reality (AR), and virtual reality (VR) systems.

Micro-LEDs can be classified into two categories according to application scenarios. The first category is display applications with relatively low pixel density (PPI < 1000), which are mainly driven by thin-film transistors (TFTs) or micro-integrated circuits (Micro-ICs). This category covers small-to-medium-sized displays (such as those for smartwatches, mobile phones, and in-vehicle displays) and large-sized displays (such as those for televisions, laptops, and monitors). The second category is display applications with high pixel density (PPI > 1000), which are usually driven by complementary metal-oxide-semiconductor (CMOS) chips. These are mainly used in micro-displays for near-eye displays, such as augmented reality (AR), virtual reality (VR), and mixed reality (MR) devices [14,15].

**Figure 1:**
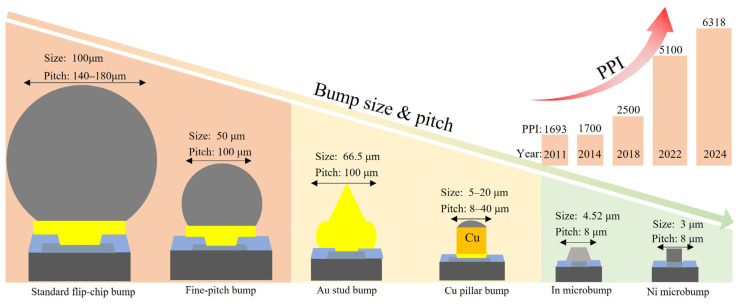
The bump size and pitch in the figure are referenced to [21,22,23,24,25], and the pixel per inch (PPI) parameter is referenced to [9,26,27,28,29].

Based on the advantage of its micron-scale size, Micro-LED can achieve a chip-integration density on the unit-area driving substrate that is typically two orders of magnitude higher than that of LCD/OLED [16]. The demand for ultra-high-density interconnection between chips and substrates has promoted the in-depth application of Flip-Chip technology. Flip-Chip technology uses a bump array to replace traditional wire bonding. Wire bonding technology is inherently limited by its physical connection method, which restricts I/O ports to the periphery of integrated circuit (IC) edges [17]. In contrast, Flip-Chip bump technology utilizes solder ball arrays to enable direct interconnections between the die and substrate, fully leveraging the entire chip surface area [17,18]. This technology can be traced back to the 1960s. It was commercialized by IBM through the Controlled Collapse Chip Connection (C4) process [19,20]. Its core process involves fabricating solder bumps on the active surface of the chip. Bumps are tiny metallic structures in integrated circuit packaging, serving to establish electrical and mechanical interconnections between the chip and the substrate or among chips. These bumps are typically formed on the Under Bump Metallurgy (UBM) of the chip pads via electroplating and evaporation processes. Figure 1 presents a preliminary exploration of historical trends in bump size and pitch, suggesting a potential correlation between the gradual miniaturization of bump structures and improvements in pixels per inch (PPI). This observation highlights the possible interplay between advancements in bump-fabrication techniques and display resolution optimization. Subsequently, direct interconnection with the substrate electrodes is accomplished through Flip-Chip bonding.

In 1996, Philips Lumileds successfully developed an LED device with a Flip-Chip structure, which marked a breakthrough application of this technology in the optoelectronic field [30]. In terms of optical performance, by eliminating the light-shading effect of bonding wires and the light-absorption loss of metal electrodes, the Flip-Chip structure increases the light-extraction efficiency of LEDs by more than 30% [31]. With the continuous trend of consumer electronics devices towards high performance and miniaturization, the interconnection density of Micro-LED displays has exceeded the order of 10^4^ bumps/mm^2^, reaching 1.56 × 10^4^ bumps/mm^2^ (3175 PPI) [24] or even higher [28,29]. Consequently, the bump pitch has entered the sub-10 μm era. Based on the mentioned technical advantages, over the past six decades, Flip-Chip technology has played a pivotal role in driving technological advancements in the field of advanced packaging.

As the core structure for device interconnection, Micro-LED bumps are micron-scale metal protrusions formed on the surface of the chip or the driving backplane. These micron-scale metal protrusions are typically formed on the surface of the chip or the driving backplane through processes such as evaporation or electroplating. The materials used include Cu, Sn, In, Ni, Au, etc., to achieve electrical interconnection and mechanical support between the chip and external circuits. The design and manufacturing of Micro-LED bumps need to comprehensively consider the following key aspects:

1. Patterning process: Micron-scale graphic templates are formed on the substrate surface through photolithography technology (for example, using positive photoresist and defining the bump positions through exposure and development), controlling the position accuracy of the bumps within ±0.5 μm.
2. Metal deposition technology: The optimal deposition process is selected according to different metal materials.
3. Reflow soldering for forming: Thermal reflow is carried out in an inert gas or reducing environment, enabling the molten metal to form spherical bumps through surface tension and reducing the porosity to less than 5% [24,32,33].

Currently, the fabrication of high-density Micro-LED bumps still faces multiple challenges. For instance, the height, shape, and composition of micrometer-scale bumps need to be strictly consistent [34]. The difference in the coefficient of thermal expansion (CTE) between the Micro-LED chip and the substrate generates stress during thermal cycling [35,36], leading to bump fracture (the smaller the bump size, the greater the fracture risk) or interface delamination. To address these technical bottlenecks, multi-dimensional coordinated efforts are required, including the optimization of the material system, process innovation, and equipment upgrading. Breakthroughs in these areas play a decisive role in improving the mass-production yield of Micro-LED and enhancing its cost-competitiveness. They are the core technical support for the commercial application of Micro-LED.

**Figure 2:**
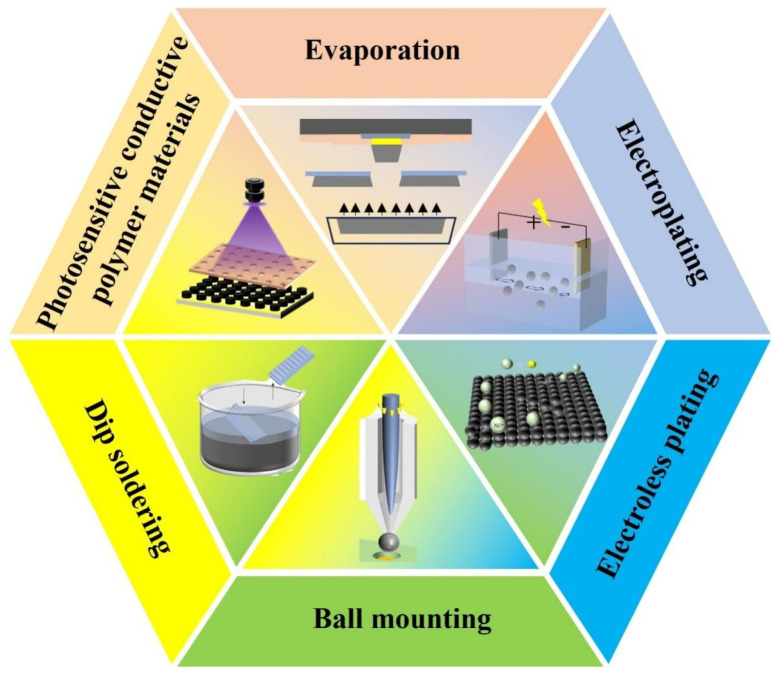
Six methods for preparing bumps.

Previous scholars have conducted systematic reviews on the research progress in Micro-LED display technology [14,37,38,39,40,41,42,43], Micro-LED display chip manufacturing, transfer, and color conversion [44,45,46,47,48], flexible Micro-LED fabrication technologies and their applications [49], research advances in pixel driving technologies for Micro-LED displays [50], and comparative studies between Micro-LED display technology and other display technologies [16,51,52,53]. However, systematic reviews focusing on bump-fabrication processes in the field of Micro-LED display technology remain extremely scarce. This paper presents a systematic technical review of bump-fabrication processes in Micro-LED technology. The article analyzes the process characteristics and technical bottlenecks of various bump-preparation technologies. Section 2 and Section 3 introduce traditional bump fabrication techniques in the Micro-LED field, including evaporation and electroplating. Section 4 elaborates on novel bump-preparation technologies such as electroless plating, ball mounting, dip soldering, and photosensitive conductive polymer materials (PCPM). Section 5 provides a critical analysis of the preparation methods for bump materials. The schematic diagrams and process comparisons of the six techniques are shown in Figure 2 and Table 1, respectively. The bump sizes and pitches in Table 1 are the minimum parameters documented in this review. Finally, the paper prospects the future development of Micro-LED bump-fabrication technologies to advance the large-scale commercialization of Micro-LED display technology in high-resolution and flexible applications.

## 2. Evaporation

**Figure 3:**
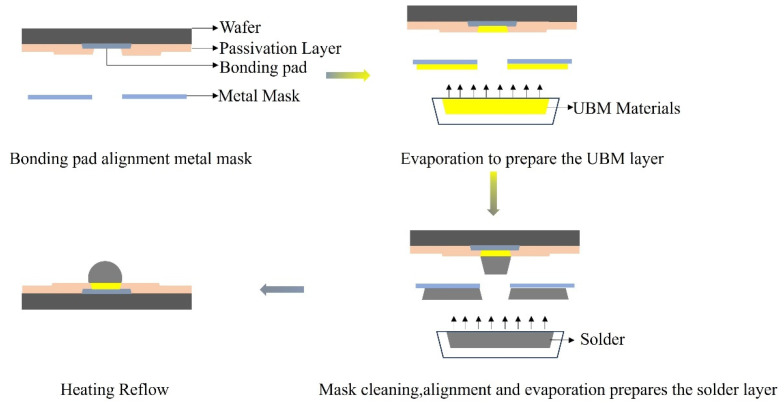
Schematic diagram of the evaporation process.

The evaporation process facilitates selective deposition of the UBM layer and solder materials through a photolithographically defined metal mask. Alternatively, a photoresist can also be employed as a masking layer for pattern definition, enabling higher-precision microstructure fabrication through photolithography processes. The UBM, serving as a critical transition layer between the bump and the pad, has a dual role. Firstly, it acts as an adhesion substrate for solder deposition, typically with a thickness ranging from 0.5 to 2 μm. Secondly, a diffusion-blocking mechanism, such as a TiW/Cu-laminated structure, restrains the migration of solder components into the chip. Its deposition process can be carried out using physical vapor deposition (PVD)/chemical vapor deposition (CVD) techniques like evaporation, sputtering, or electroplating [54]. The processes for preparing bumps and the UBM by the evaporation process are illustrated in Figure 3. The procedures for preparing bumps by magnetron sputtering deposition are similar to those of evaporation deposition. Evaporation deposition has become the top choice for bump fabrication due to its high deposition rate, compatibility with low-melting-point materials, and cost-effectiveness. Although magnetron sputtering demonstrates excellent performance in terms of uniformity and adhesion, its slow speed and limited local-forming ability make it more suitable for scenarios where a uniform thin film is required, rather than for structures like bumps that demand rapid local accumulation [55]. Although traditional lead-based solders have significant advantages in terms of interface reliability (such as thermal fatigue resistance) and mechanical strength [56,57], the cumulative neurotoxic effects of lead in organisms and its persistent pollution characteristics in the ecosystem have prompted major economies around the world to gradually replace the traditional Sn-Pb solder system with lead-free technology routes, thus promoting the industrial application of new interconnection materials.

### 2.1. Evaporation Process and Material Selection

**Figure 4:**
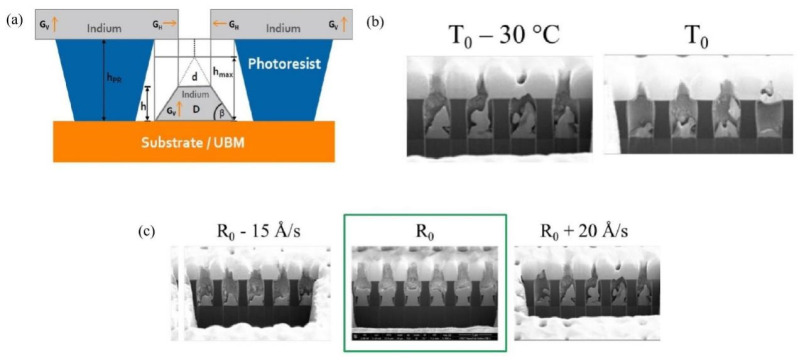
(**a**) h_PR_ represents the photoresist height, h represents the indium bump height, h_max_ represents the maximum possible bump height, D represents the diameter of the photoresist opening (i.e., the bottom diameter of the bump), and d represents the top diameter of the bump. (**b**) Cross-sectional SEM image displaying the impact of substrate temperature decrease on the deposited indium bumps. (**c**) Cross-sectional SEM images illustrate the variation of indium bumps deposited at different evaporation rates. (**a**–**c**) Reproduced with permission [60]. Copyright 2018, SPIE Publication.

**Figure 5:**
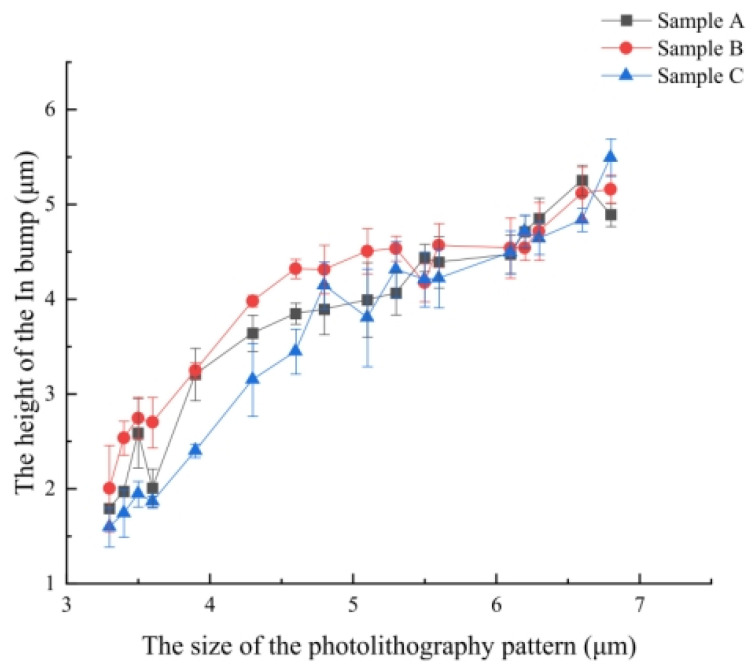
Relationship between the dimensions of the photolithography pattern and the height of the In bump. Reproduced with permission [61], Copyright 2024, IOP Publishing.

In the field of high-density interconnection of Micro-LED devices, indium (In) has become the preferred solder material for evaporation/sputtering processes over the past decade due to its unique physical properties. The most crucial reason is that its low melting point of 156 °C can significantly reduce the thermal stress caused by the mismatch in the coefficient of thermal expansion between the driving substrate and the Micro-LED chip [58,59]. Although there are occasional research reports on Sn-based solders in the literature, their vapor pressure at the same temperature is lower than that of indium, leading to a relatively slower evaporation deposition rate [18]. In addition, the relatively high melting point of Sn (231.9 °C compared to 156.6 °C of indium) can cause a more significant thermal expansion mismatch during the bonding process. In 2011, Day et al. employed the indium bump-evaporation process on the Micro-LED array and achieved Flip-Chip interconnection with a silicon-based CMOS substrate through thermocompression bonding (TCB) [26]. They achieved a technological breakthrough by developing a novel semiconductor-based green microdisplay with verified video graphics image transmission capability, marking a significant advancement in microdisplay engineering. This research demonstrated the morphology of indium bumps with a diameter of 6 μm and a bump pitch of 15 μm. Although the thermal stability test showed that the optical output power decreased by 10% under high-temperature conditions (100 °C) due to the aggravated hot carrier effect, the breakthrough achievement of the 15-μm bump pitch provided important technical references for the subsequent development of electrode interconnection technology for high-density Micro-LED displays. When discussing the progress of indium bump-evaporation technology, an important paper published by Manasson et al. in 2018 cannot be overlooked [60]. They investigated the influence of key process parameters such as evaporation rate and substrate temperature on the formation of indium bumps. Their goal was to achieve a high-density interconnection array with a small pixel pitch for the next-generation infrared focal plane array (FPA) detectors, which is also of great significance for the interconnection technology of high-precision optoelectronic devices such as Micro-LEDs. The research shows the dynamic changes of the thin film during the indium evaporation process through Figure 4a, revealing the growth mechanism of indium bumps within the patterned through-holes of the photoresist and the process of through-hole opening closure. The ratio of the vertical growth rate (G_V_) to the horizontal growth rate (G_H_) reflects the tendency of through-hole closure. Experiments indicate that when the substrate temperature decreases, the G_V_/G_H_ ratio increases significantly, as shown in Figure 4b. This phenomenon is related to the enhanced anisotropy of thin-film growth caused by the reduced surface mobility of indium atoms at low temperatures. However, low temperatures can also exacerbate the surface roughness of the thin film and may cause the photoresist to crack. In addition, the influence of the evaporation rate on through-hole closure and surface roughness is non-linear. The research determined the optimal evaporation rate R_0_ through multiple sets of experiments, as shown in Figure 4c. At this rate, the through-hole opening is closer to circular and has a larger area, thus forming a more uniform indium bump morphology. These findings provide crucial guidance for optimizing the indium bump-preparation process. For example, in infrared focal plane arrays, by balancing the substrate temperature and determining the combined parameters of the optimal deposition rate, a bump array with high uniformity and small pitch can be achieved, significantly improving the shear strength of indium bumps in Flip-Chip interconnection and the device reliability. In 2024, Jiang et al. demonstrated indium bump arrays with a pitch of 8 μm via optimized evaporation-plating protocols, addressing critical challenges in miniaturization [61]. However, the industrial preparation of such micro-bump arrays still faces challenges. If the lithography aperture is too small, the horizontal growth of indium may block the vias, resulting in insufficient bonding height. If the aperture is too large, it is likely to cause short-circuits between bumps. As shown in Figure 5, Jiang et al. systematically studied the influence of the lithography pattern aperture ranging from 3.0 to 7.0 μm on the height of indium bumps under photoresist thicknesses of 3.8 μm (Sample A/B) and 4.8 μm (Sample C), revealing the significant regulatory effect of the lithography pattern size on the bump height. This provides a basis for the selection of key parameters for the preparation of high-precision indium bumps on Micro-LED chips.

### 2.2. Reflow Process Optimization

When using the evaporation/sputtering method to prepare high-quality indium bumps for bonding, in addition to controlling the substrate temperature and evaporation rate, it is also necessary to eliminate the influence of indium oxide through the reflow process. Since the surface of indium bumps exposed to air will form an indium oxide layer with high melting point and high surface energy, which makes it difficult to melt during bonding and affects the bonding quality, it is necessary to carry out reflow treatment in a reducing atmosphere such as formic acid or hydrogen to remove the oxide layer. However, the strong corrosiveness of formic acid vapor and the flammable and explosive characteristics of hydrogen make this process a significant safety hazard, and risks must be avoided through strict safety protection measures and standardized operation procedures [55]. Ji et al. (2022) [32] and Zhu et al. (2023) [33] have developed two safer and lower-cost methods.

#### 2.2.1. Reflow in Glycerol

**Figure 6:**
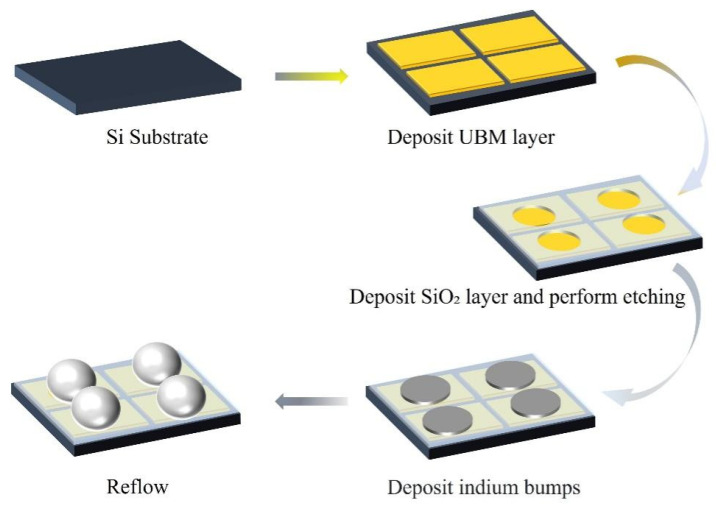
The manufacturing process of indium bumps.

Ji et al. carried out the reflow of indium bumps in glycerol [32]. The experimental process is shown in Figure 6. In the first step, a Ni/Au stacked layer was prepared as the UBM on the Si substrate by electron-beam evaporation. In the second step, a SiO_2_ layer was deposited by plasma-enhanced chemical vapor deposition (PECVD), and 5 μm contact holes were etched by inductively coupled plasma (ICP). In the third step, indium was deposited on the UBM by electron-beam evaporation with the help of a photoresist mask and then patterned by liftoff. In the fourth step, the reflow was conducted in glycerol. Taking advantage of the property that the surface tension of molten indium is greater than its surface energy, the pancake-shaped bumps were transformed into spherical ones. Ji’s research team successfully prepared a 10 × 10 indium bump array with a pitch of 40 μm on the Si substrate. It was observed that as the reflow time increased, the bump diameter decreased from 19.4 μm to 14.9 μm. The effects of reflow temperature and time on reliability were analyzed through shear tests. It was found that when the reflow time was 180 s and the temperature was 200 °C, the average shear strength of indium micro-bumps reached its peak and then decreased as the parameters increased. Combined with SEM fracture surface analysis, it was proven that the change in shear strength is directly related to the evolution of the intermetallic compound (IMC) at the Au/In interface. In the 0–180 s stage, the formation of IMC and the small grain size enhanced the interfacial connection strength. However, after more than 180 s, the increase in IMC thickness and grain coarsening (in line with the Hall–Petch [62,63] effect) led to a decrease in strength. According to Hall–Petch’s description:

(1) $$\sigma_{s}=\sigma_{0}+{kd}^{-\frac{1}{2}}$$

where $\sigma_{s}$ is the yield stress, $\sigma_{0}$ is k material constants, and d is the grain size.

#### 2.2.2. Reflow in Flux

Zhu et al. prepared indium micro-bumps with a high degree of sphericity in an atmospheric environment through a wet reflow process [33]. This process involves a two-step lithography approach. In the first lithography step, a titanium (Ti) solder mask with a diameter of 18 μm was defined. In the second step, indium deposition holes with a diameter of 55 μm were defined. High-purity indium (99.999%) was then deposited by magnetron sputtering at a deposition rate of 0.06 μm/min. During the reflow process, a comparison was made between a liquid flux (WS-807, Hidaka Engineering Co., Ltd., Ueda, Japan) and a solid flux (WF-6317, SMIC, Shanghai, China). It was found that due to its low viscosity, the liquid flux significantly reduced the interference with the surface tension of molten indium. As a result, spherical micro-bumps with a diameter of approximately 20 μm and a height deviation of ±0.87 μm were formed, with a yield exceeding 99.7%. In contrast, the high viscosity of the solid flux caused the bumps to take on a hill-like shape. The peak of the reflow temperature curve was set at 168 °C, which is higher than the melting point of indium (156.5 °C). Thermal stress was avoided by optimizing the pre-heating and cooling stages. Interface analysis indicates that the Au-In reaction only generates the AuIn_2_ intermetallic compound (verified by EDS), and no other IMC phases are detected. The thermal aging experiment (at 70 °C for 0–400 h) shows that the IMC grains gradually coarsen as the aging time prolongs. However, due to the depletion of the Au layer, the growth tends to stagnate. The shear strength drops from approximately 40 MPa initially to 25 MPa after 400 h. Meanwhile, the fracture mode transforms from ductile (fracture inside the bump) to brittle (interface fracture).

Both Ji’s team and Zhu’s team replaced the traditional reflow in a formic acid or hydrogen environment with a reflow using glycerol and liquid flux. This approach is safer and more cost-effective and can produce high-quality micro-bump arrays. Their research is expected to promote the commercial application of advanced packaging technologies such as Micro-LED, accelerating the development process of related industries.

#### 2.2.3. Reflow on Micro-LED Chips

**Figure 7:**
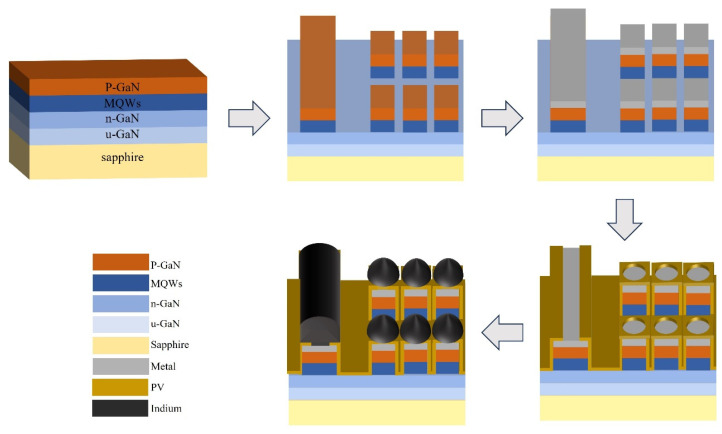
The growth process of indium bumps.

**Figure 8:**
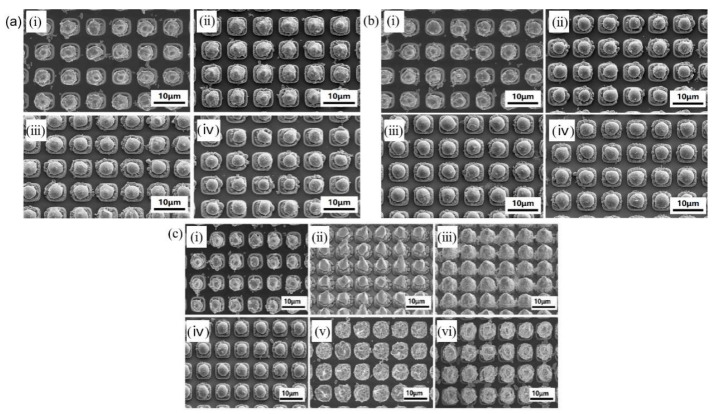
(**a**) SEM images showing the morphology of the indium bumps which were reflowed at different temperatures for 90 s are as follows: (**i**) without reflow, (**ii**) 230 °C, (**iii**) 250 °C, and (**iv**) 270 °C. (**b**) SEM images presenting the morphology of the indium bumps which were reflowed at 270 °C for different durations are shown as follows: (**i**) 0 s, (**ii**) 90 s, (**iii**) 180 s, and (**iv**) 270 s. (**c**) SEM images showing the morphology of the indium bumps after undergoing reflow at 270 °C for 90 s are presented as follows: (**i**) sample A prior to reflow, (**ii**) sample A subsequent to reflow, (**iii**) sample B before reflow, (**iv**) sample B after reflow, (**v**) sample C before reflow, and (**vi**) sample C after reflow. (**a**–**c**) Reproduced with permission [24], Copyright 2025, Elsevier.

In 2025, Yang et al. broke through the limitations of traditional planar substrate research and for the first time carried out research on the indium bump reflow process combined with thermal evaporation technology on Micro-LED chips, targeting indium bumps with a diameter of about 5 μm and a pixel pitch of 8 μm [24]. The preparation process of indium bumps is shown in Figure 7. In the first step, Micro-LED materials are epitaxially grown on a multiple quantum well (MQW) layer and a p-GaN layer. In the second step, inductively coupled plasma (ICP) dry etching technology is employed to create a mesa structure on the epitaxial layer, thereby defining the light-emitting area of the Micro-LED chip. The large area on the far left of the top serves as the common cathode (n-electrode), while the rest are p-electrodes. In the third step, electron-beam evaporation technology is used to deposit p-type and n-type electrodes of equal height to meet the requirements of subsequent bonding. In the fourth step, a silicon dioxide (SiO_2_) insulating layer is deposited, and windows are opened to achieve an electrical connection between the n-electrode and the p-electrode. In the fifth step, the negative photoresist is spin-coated, and after exposure and development, indium bumps are thermally evaporated. In the sixth step, the indium bumps are placed in a formic acid environment for reflow and then subjected to thermocompression bonding. They analyzed the effects of formic acid reflow temperature, time, mesa height, and metal thickness on indium bumps in their research on the reflow process. As shown in Figure 8a, at 230 °C, the indium bumps only show a weak spheroidization trend (the average size is fine-tuned from 4.77 μm to 4.76 μm). However, at 250 °C and 270 °C, the spheroidization is significant, with fewer surface protrusions and a significantly narrower particle-size distribution (the average sizes decrease to 4.47 μm and 4.52 μm, respectively). The particle-size distribution is the narrowest at 270 °C. Figure 8b shows that at 270 °C, indium bumps complete spheroidization and the particle-size distribution narrows after 90 s of reflow (the average size decreases from 5.06 μm to 4.74 μm). At 180 s, the particle size further shrinks to 4.52 μm, but the morphology remains stable. There is no significant change in size at 270 s, indicating that the oxide layer may not be completely reduced at 90 s, and the time needs to be optimized to form a uniform spherical structure. Figure 8c compares three Micro-LED samples with different structures (the mesa heights of Samples A, B, and C are 1.5 μm, 1.5 μm, and 2.5 μm, respectively, and the metal thicknesses are 0.2 μm, 2.0 μm, and 0.2 μm, respectively). Under the same lithography process and reflow conditions of 270 °C/90 s, an increase in mesa or metal height leads to a deterioration of the indium reflow effect, making it impossible to form a spherical structure and resulting in a more irregular bump morphology.

In the Micro-LED field, although many researchers use the evaporation process to prepare micro-bumps, their research mainly focuses on aspects such as bonding technology, luminous brightness regulation, pixel density optimization, and enhancement of color-rendering performance, rather than the growth mechanism of the bumps themselves. The bump parameters are shown in Table 2.

### 2.3. Challenges and Limitations

As can be gleaned from the aforementioned reports, in the preparation of micro-bumps, the evaporation-deposition method is extensively utilized, thanks to its remarkable advantages of micron-scale dimensional accuracy control, highly dense thin-film formation, and a low defect rate. Notably, through precise manipulation of evaporation parameters, the sequential deposition of multiple layers of heterogeneous materials can be accomplished, rendering it particularly adept at producing small-batch, high-precision bumps [55]. However, this technology is confronted with two pivotal bottlenecks. Firstly, production efficiency is constrained. High-temperature evaporation necessitates the maintenance of a complex vacuum environment. Consequently, the quantity of substrates loaded in a single run is limited by the size of the vacuum chamber. Additionally, the deposition rate generally falls below 20 Å/s, making it arduous to meet the demands of wafer-scale mass production. Secondly, the overall cost of the equipment is exorbitant. The equipment incorporates a high-precision electron-beam heat source, an ultra-high-vacuum system driven by a molecular pump unit (where maintenance costs account for 30–40% of the total cost), and a quartz-crystal film-thickness monitoring module with an accuracy of ±0.1 Å. This reliance on professional teams for equipment procurement and subsequent maintenance further escalates the process cost.

## 3. Electroplating

### 3.1. Fundamentals of Electroplating for Bump Fabrication

**Figure 9:**
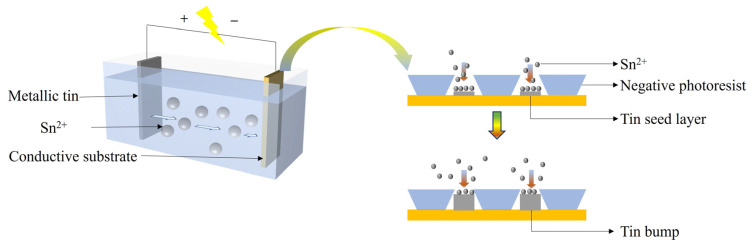
Process of electroplating to grow Sn bumps.

Compared with the evaporation method, the electroplating method exhibits remarkable advantages in bump fabrication. Electroplating leverages electrochemical reduction mechanisms to enable synchronous multi-surface deposition, thereby enhancing production efficiency through scalable substrate immersion [72]. For example, when electroplating and growing Sn bumps in the field of Micro-LED (the process is shown in Figure 9), the anode metal is oxidized to release ions into the electrolyte, and the metal ions migrate to the cathode surface driven by the electric field to gain electrons and undergo a reduction reaction (as shown in Equations (2) and (3)), thus forming a uniform bump structure through layer-by-layer stacking. This process not only has a fast deposition rate and can meet the requirements of wafer-level mass production, but also significantly reduces the equipment cost [18]. The electroplating system only needs to be equipped with a basic electrolytic cell, a conventional power supply, and electrode components. It doesn’t require the high-vacuum environment maintenance device or the precise temperature control module needed in the evaporation method. The simplified overall structure significantly reduces the equipment-purchase and maintenance costs. Moreover, the operation threshold is also notably lowered, providing a better solution for the low-cost and large-scale manufacturing of high-density interconnection structures.

(2) $$\mathrm{anodic}\;\mathrm{reaction:}\;Sn={Sn}^{2+}+2e^{-}$$

(3) $$\mathrm{cathodic}\;\mathrm{reaction:}\;{Sn}^{2+}+2e^{-}=Sn$$

IBM’s C4 (Controlled Collapse Chip Connection) technology has undergone decades of iterations, and its core process has fundamentally shifted from evaporation to electroplating [20,30]. In the early days, when the evaporation process was used to fabricate C4 bumps, the metal mask showed a deficiency in mechanical stability when dealing with the increasing interconnection density (for example, mask deformation led to bump-positioning deviation). This technological bottleneck directly promoted the industrial application of the electroplating process [18].

**Figure 10:**
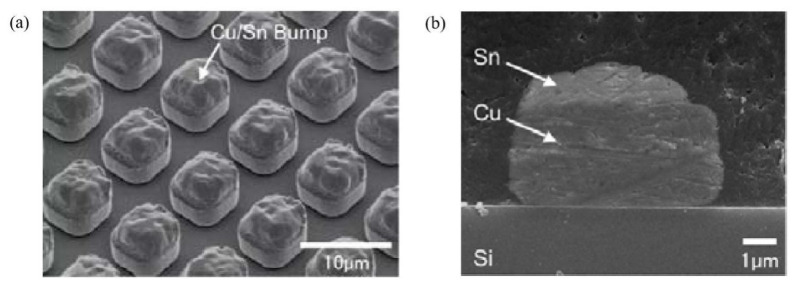
(**a**) Morphology of square Cu/Sn bumps. (**b**) Cross-sectional SEM images of Cu/Sn bumps. (**a**,**b**) Reproduced with permission [73], Copyright 2009, Japan Society of Applied Physics.

In 2009, Ohara et al. proposed a composite electroplating-evaporation strategy [73]. By first electroplating copper bumps on the pads as a support structure and then evaporating a Sn layer, Cu/Sn composite micro-bumps were formed. They successfully reduced the bump pitch to 10 μm and applied it to three-dimensional chip stacking. This technological breakthrough resonates with the requirement of achieving high-density interconnection below 50 μm in the Micro-LED field, as both need to precisely control the bump morphology and the consistency of spatial distribution at the micrometer scale. As shown in Figure 10a, the Cu/Sn composite micro-bumps fabricated by Ohara et al. have a square cross-section with a side length of approximately 5 μm. The overall average height is 5.0 μm (3 μm for the Cu layer and 2 μm for the Sn layer), and the height-fluctuation range is controlled within ±3%. According to the uniformity evaluation equation (Equation (4)) proposed by Tian et al. [59], researchers in the field of pixel detectors, the calculated bump uniformity is 3%, which indicates excellent height consistency.

Although the research object of Tian’s team is pixel detectors, their systematic research results in aspects such as electroplating process parameters (e.g., the influence of pulsed current waveforms on deposition kinetics), microstructure control (the correlation between grain orientation and defect density), and uniformity quantification methods (statistical model construction) provide cross-domain technical references for the electroplating technology of ultra-fine pitch bumps in Micro-LEDs (e.g., suppression of the morphological anisotropy of indium bumps and optimization of grain boundary distribution). In terms of the morphological control of indium bumps, direct-current electroplating (waveform i, 10 mA/cm^2^) leads to coarse grains and central hollow defects due to current concentration at the photoresist openings. In contrast, unipolar pulsed electroplating (waveforms ii–iv) refines the grains and improves the surface flatness through periodic deposition interruptions. However, the morphological differences among unipolar pulses with different parameters are limited. Bipolar pulses (waveforms v–vi) introduce reverse pulses (for example, waveform v: forward and reverse current densities are 50 mA/cm^2^, forward duration is 1.5 ms, reverse duration is 0.5 ms, off-time is 3 ms, frequency is 200 Hz; waveform vi: forward and reverse current densities are increased to 100 mA/cm^2^ and the off-time is extended to 8 ms, frequency is 100 Hz), taking advantage of the preferential dissolution effect of the anodic cycle on the protruding parts of the bumps. Microstructural analysis shows that under all conditions, the grain size is larger than that in direct-current electroplating and there are no impurities. EDX confirms the pure indium composition. Regarding the influence on the height uniformity of indium bumps, Tian’s team optimized the height uniformity from 19.65% in direct-current electroplating to 14.3–15.2% in unipolar pulsed electroplating and 13.6–14.07% in bipolar pulsed electroplating.

(4) $$Uniformity=\frac{\mathrm{Max:}BumpHeight-\mathrm{Min:}BumpHeight}{2AverageBumpHeight}\times 100\%$$

In the field of Micro-LED, as early as 2009, Liu et al. had already realized a technical solution for bonding GaN-based Micro-LED chips by electroplating Cu bumps on a CMOS substrate [74]. Most of the research on electroplated bumps still draws on studies from other electronic packaging fields. For example, Zhang et al. referred to the successful cases of Au-free metal bonding in fields such as 3D chip stacking [75]. They grew Cu/Sn bumps on Micro-LED chips through the electroplating process and successfully achieved Au-free metal bonding, further reducing the cost of Micro-LED chips. In 2023, Jiang et al. also used this technology to reduce the cost of manufacturing Micro-LED chips [76]. However, during the process of growing bumps by electroplating, the uniformity of bump height has always been a difficult problem. This problem is widespread in various industries, which is also one of the reasons why Ohara et al. chose to electroplate Cu first and then evaporate Sn.

### 3.2. Process Parameter Optimization

In the field of Micro-LED, if uniform metal bumps cannot be grown, it will have an adverse impact on the high-yield bonding process [59]. In 2023, Luo et al. successfully electroplated Cu/Sn bumps with ultra-fine pitch and high uniformity by studying the effects of the anode-cathode distance, plating solution stirring rate, and current density on the morphology and uniformity of electroplated tin bumps [23].

#### 3.2.1. Anode–Cathode Spacing Optimization for Bump Uniformity

**Figure 11:**
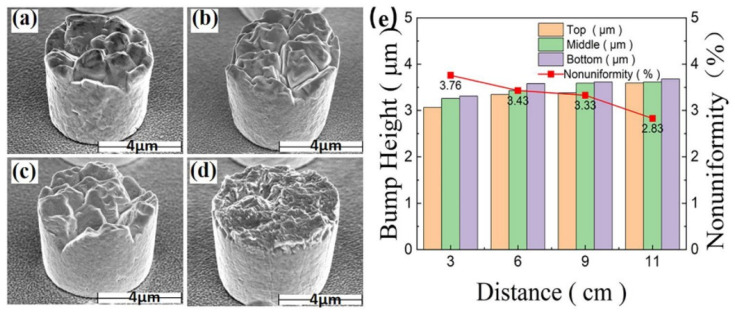
(**a**–**d**) SEM images of plated Sn bumps with cathode–anode spacing of 3 cm, 6 cm, 9 cm, and 11 cm, respectively. (**e**) Histograms of height and uniformity values of tin bumps grown at different cathode and anode spacings. (**a**–**e**) Reproduced with permission [23], Copyright 2024, Springer Nature.

The effect of the anode–cathode distance on the bump morphology and uniformity is shown in Figure 11. When the anode–cathode distance increases to 11 cm, the uniformity of Sn bumps is the best. As the anode–cathode distance increases, the electrolyte potential distribution becomes more uniform, resulting in a smoother surface and a more uniform height of Sn bumps. However, the distance is not the larger the better, as an overly large distance will also reduce the electroplating efficiency. This is because the distance affects the current density distribution and the degree of cathodic polarization. An appropriate distance can make the current densities near and far from the cathode close to each other, thereby improving the bump uniformity.

#### 3.2.2. Stirring Rate Modulation for Enhanced Mass Transfer and Surface Quality

**Figure 12:**
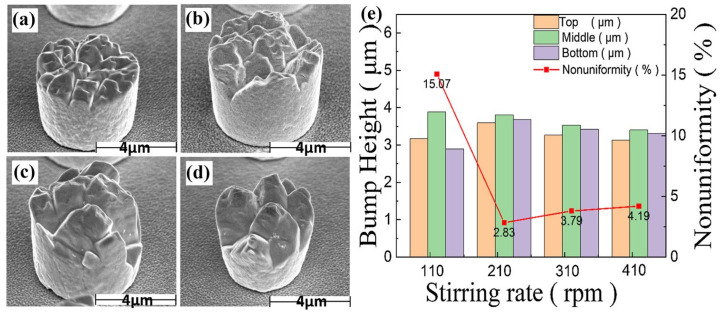
(**a**–**d**) SEM images of plated Sn bumps with stirring rates of 110 rpm, 210 rpm, 310 rpm, and 410 rpm, respectively. (**e**) Histograms of height and uniformity values of tin bumps grown at different stirring rates. (**a**–**e**) Reproduced with permission [23], Copyright 2024, Springer Nature.

The influence of the stirring rate of the plating solution on the morphology and uniformity of the bumps is shown in Figure 12. It can be seen that the uniformity of the Sn bumps is the best when the stirring rate is 210 rpm. The reason why stirring the plating solution can improve the uniformity of the Sn bumps is that many additives in the plating solution are organic macromolecules or high-molecular compounds with large ionic radii, which have low mobility. Without the promotion of stirring, the additives consumed in the cathode adsorption layer are difficult to replenish in time. Sufficient stirring force can increase the convective mass-transfer rate, compensate for the problem of hindered electrode reactions caused by insufficient spontaneous mass transfer, enable the timely replenishment of the consumable liquid at the cathode interface, and reduce pinholes and pits. However, an excessively high stirring rate will reduce the concentration-difference polarization at the cathode, resulting in rough Sn crystallization and non-uniform surface morphology.

#### 3.2.3. Current Density Balancing for Polarization and Deposition Control

**Figure 13:**
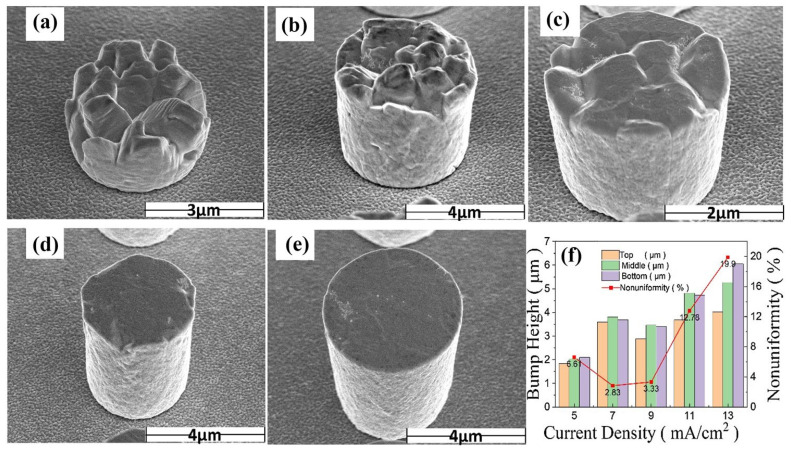
(**a**–**e**) SEM images of plated Sn bumps with current densities of 5 mA/cm^2^, 7 mA/cm^2^, 9 mA/cm^2^,11 mA/cm^2^, and 13 mA/cm^2^, respectively. (**f**) Histograms of height and uniformity values of tin bumps grown at different current densities. (**a**–**f**) Reproduced with permission [23], Copyright 2024, Springer Nature.

The influence of current density on the morphology and uniformity of electroplated tin bumps is shown in Figure 13. Experiments have found that the uniformity of Sn bumps is the best when the current density is 7 mA/cm^2^. As the current density increases, the surface of Sn bumps changes from rough to smooth, but the uniformity deteriorates at an excessively high current density. Generally speaking, a higher current density helps to improve production efficiency, while a lower current density allows for more controllable growth of the deposit. Moderately increasing the current density can enhance the cathodic polarization effect, thereby improving the efficiency and uniformity of electroplating. However, an excessively high current density will make the electrolyte potential distribution uneven and affect the height uniformity of the bumps.

#### 3.2.4. Semi-Additive Plating (SAP) Process

**Figure 14:**
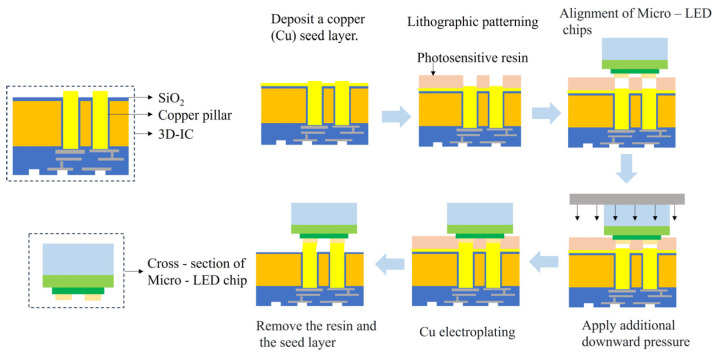
Process of electroplating to connect Micro-LED and 3D-IC.

In 2023, Shen et al. proposed a very creative idea of growing Cu bumps through SAP (Semi-additive Plating process) electroplating [77]. The electroplating process is shown in Figure 14. The first step: deposit a seed layer on the 3D-IC. The second step: spin-coat the photosensitive resin on the 3D-IC and expose the electrodes at the top of the 3D-IC through patterned photolithography. The third step: pick up the Micro-LED chip and precisely place it in the opening position. The fourth step: apply additional extrusion to prevent the potential tilt of the Micro-LED chip. The fifth step: electroplate and grow Cu. The sixth step: peel off the photosensitive resin. We think that since the fourth step adds extra pressure to prevent tilting, if the added extra pressure is too large, as the height of the Cu pillar continues to increase, the electroplating solution may not be able to be replenished into the through-hole in a timely manner in the later stage of electroplating. This may lead to the insufficient height of the electroplated Cu pillar, or the top of the grown Cu pillar may not be able to fully contact the electrodes of the Micro-LED chip. This situation may lead to an increase in resistance and unstable connections.

### 3.3. Electroplating Technology Improvement

**Figure 15:**
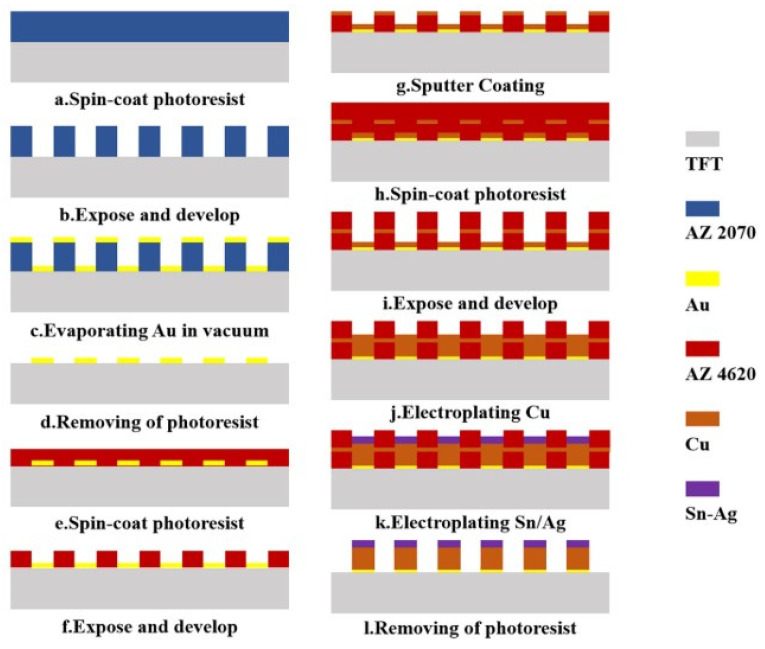
Schematic diagram of the Double-Layer Photoresist Structure Electroplating process. Reproduced with permission [78], Copyright 2025, Elsevier.

In the past, during the process of growing bumps by electroplating, after the bumps were grown, it was necessary to use etching to remove the excess seed layer to prevent the seed layer from connecting the anode and cathode of the electrode and causing a short circuit. However, the etching operation would cause a certain degree of damage to the circuit of the driving substrate. In 2024, Tang et al. proposed the Double-Layer Photoresist Structure Electroplating (DPSE) electroplating scheme, that is, a scheme of adding the seed layer in the middle of the photoresist [78]. The specific process flow is shown in Figure 15. First, spin-coat a negative photoresist (AZ NLOF2070, MicroChemicals GmbH, Ulm, Germany) with a thickness of about 4 μm on the surface of the TFT backplane. After exposure by a UV lithography machine (SUSS Micro Tec MA/BA6 Gen4, Garching, Germany ), use oxygen plasma treatment to remove the residual photoresist in the lithography holes. Then, vacuum-deposit a gold layer with a thickness of about 25 nm on the patterned surface. Finally, use a stripping solution to remove the AZ 2070 photoresist. Then, spin-coat an acid-resistant positive photoresist (AZ P4620) with a thickness of about 2 μm on the surface of the TFT backplane. After exposure and development, bake it to reduce the residual solvent in the photoresist. Then, sputter a copper seed layer with a thickness of about 50 nm. Next, spin-coat a second layer of AZ P4620 with a thickness of about 6 μm and perform secondary exposure and development to form holes of about 16.5 μm × 10 μm at the electrodes of the TFT backplane to facilitate the flow of the electroplating solution and electrochemical reactions. Finally, electroplate and grow Cu/78 wt% Sn–22 wt% Ag bumps. The reason for choosing to electroplate SnAg bumps is that, compared with Sn bumps, SnAg eutectic bumps have a lower melting point and are more suitable for the bonding process. Since the Cu seed layer is on the first layer of AZ P4620, it is only necessary to put the TFT into acetone or a stripping solution to remove the Cu seed layer. Moreover, Tang et al. reduced the uniformity of Cu/SnAg bumps to 2.266% by adjusting parameters such as current density and the distance between the positive and negative electrodes.

### 3.4. Challenges and Limitations

Electroplating demonstrates significant advantages, including relatively low cost and the capability for batch processing of multiple substrates simultaneously [72]. However, its predominant limitation lies in the non-uniform current density distribution across conductive substrates, which directly affects the height consistency of deposited bumps. This phenomenon primarily stems from the inherent structural characteristics of substrates, their surface states, and complex interfacial interactions with the electrolyte solution. The uneven current distribution results in differential deposition rates of metal ions across various substrate regions, consequently leading to bump-height variations [25,34]. Although Luo et al. and Tang et al. have achieved notable bump-height uniformity improvements of 2.83% and 2.26%, respectively, we posit that these results were obtained using relatively small-scale test substrates, which may mitigate the current density non-uniformity effects. For commercial-scale production involving larger substrates, current density management remains a critical technical challenge that requires fundamental resolution.

## 4. Emerging Bump-Fabrication Technology

### 4.1. Electroless Plating

**Figure 16:**
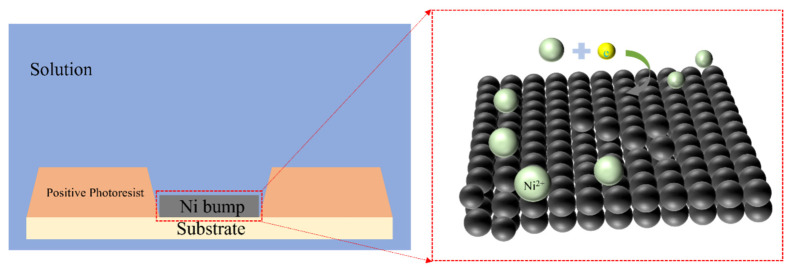
Schematic Diagram of the Electroless Ni Plating Mechanism.

Compared with the traditional electroplating process, the electroless plating process has the following three significant advantages: 1. Based on the mechanism of the autocatalytic reduction reaction, electroless plating can achieve metal deposition without relying on external current, effectively avoiding the bump-height difference caused by uneven current distribution [79]. 2. The process is not limited by the conductivity of the substrate and can be extended to the surface metallization treatment of non-conductive substrates [79]. 3. It has a better economy in terms of equipment investment, energy consumption, and raw material utilization [80]. Taking electroless Ni bumps as an example, its growth mechanism is shown in Figure 16.

**Figure 17:**
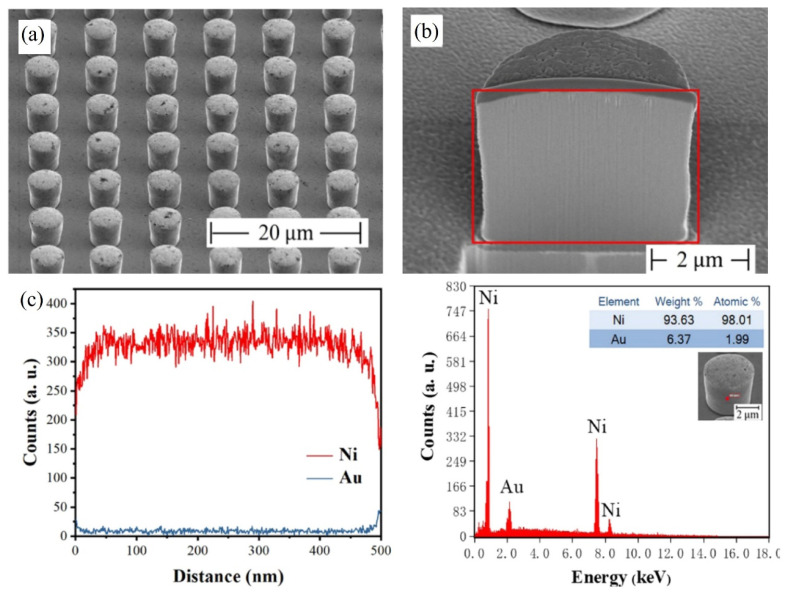
(**a**) SEM images of the Ni/Au bump arrangement. (**b**) SEM image of the cross-section of the Ni/Au bump showing no voids. (**c**) EDS line scan of the cross-section of the Ni/Au bump and EDS spectrum of the bump sidewall. Reproduced with permission [25], Copyright 2022, American Chemical Society.

The technology of fabricating bumps by electroless plating has become a research hotspot in the field of electronic packaging due to its unique process advantages. Currently, research on the material systems for electroless plating of bumps mainly focuses on metal systems such as Au [81,82], Cu [83], Ni [25,34,84], and Sn [85]. It is worth noting that in the field of Micro-LED device interconnection, there are relatively few reports on electroless plating in existing literature, and it can be regarded as a new bump-fabrication technology. In 2022, Tian et al. introduced two catalytic methods: the iron-sheet method (after photolithography, remove the photoresist at the sample edge to expose the gold layer, and attach the sample to an iron sheet with copper conductive tape) and the nickel-layer method (first sputter a 100 nm-thick nickel layer on a gold substrate, and then perform photolithography and electroplating) [25]. The experimental results show that both catalytic methods can fabricate uniform Ni/Au bump structures with a height of 4 μm and an array pitch of 8 μm. The SEM images of the bumps and EDS analysis are shown in Figure 17. The EDS energy-spectrum analysis confirms that the interior of the bumps exhibits highly densified characteristics, and no micro-holes or impurity phases are detected. This structural integrity provides a key guarantee for achieving highly reliable Flip-Chip bonding. The research results indicate that this technology shows significant technical advantages and application potential in realizing the high-density interconnection of Micro-LED chips.

**Figure 18:**
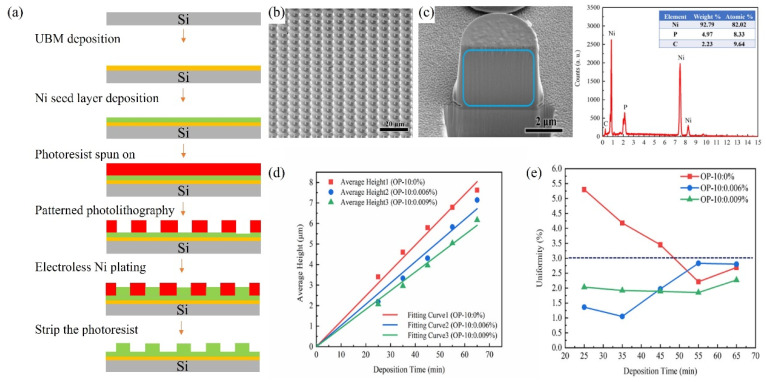
(**a**) The process flow of electroless nickel plating. (**b**) SEM image of Ni bump array. (**c**) The left image shows the SEM image of the cross-section of Ni bumps, while the right one presents the EDS spectrum analysis of the cross-section. (**d**) The change in the height of Ni bumps over time with different concentrations of OP-10 added. (**e**) The change in the height uniformity of Ni bumps over time with different concentrations of OP-10 added. (**b**–**d**) Reproduced with permission [34], Copyright 2024, Elsevier.

**Figure 19:**
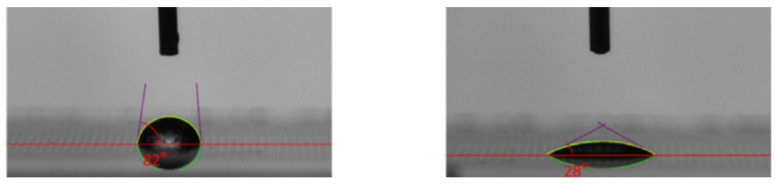
The left and right figures show the contact angles before and after O_2_ plasma treatment, respectively. Reproduced with permission [84], Copyright 2024, John Wiley and Sons.

As the bump pitch develops towards the micron scale (<10 μm), the insufficient coplanarity of the bump array (>5% height deviation) and the risk of bridging are significantly exacerbated, it seriously affects the bonding yield rate between the Micro-LED chip and the driving substrate. To address this issue, in 2024, Lu et al. successfully fabricated a nickel bump array with a pitch of 8 μm and a height of 4 μm by optimizing the parameters of the electroless nickel plating process [34]. The height uniformity of the bump array is less than 3%. The process flowchart of the electroless Ni plating is shown in Figure 18a. The specific optimized parameters are as follows: 1. Optimization of plasma treatment time: The plasma modification time was set to 7 min. Plasma treatment can improve the surface wettability of the photoresist, effectively reducing the phenomenon of hydrogen bubble retention and avoiding the problem of uneven bump morphology caused by local differences in deposition rates. 2. Regulation of surfactant concentration: When alkylphenol ethoxylates (OP) with concentrations of 0.006% and 0.009% were added, the height uniformity of the bumps was significantly improved (< 3%). As shown in Figure 18e, through comparative experiments, the optimization of the OP-10 concentration effectively suppresses the influence of the surface tension gradient on the deposition process, thus ensuring the uniformity of the bump array. The SEM and EDS analysis images are shown in Figure 18 b,c. It can be seen from the figures that the bumps are dense without holes (Ni purity > 92.79%), meeting the requirements for high-reliability Flip-Chip soldering. In the subsequent optimization research on the electroless Ni bump-fabrication process, Wang et al. significantly improved the wettability of the photoresist by introducing O_2_ plasma surface modification technology [84]. As shown in Figure 19, after 5 min of O_2_ plasma treatment, the contact angle was reduced to 28°, further reducing the retention of hydrogen bubbles. Finally, they grew a highly uniform Ni bump array on the TFT substrate, with a height uniformity of less than 1%. This result is significantly better than the uniformity index of traditional processes.

### 4.2. Ball Mounting

**Figure 20:**
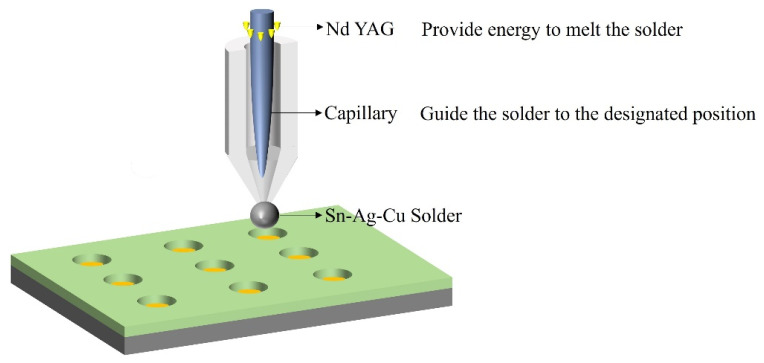
Automated bump-deposition process.

In the field of Micro-LED device interconnection, in 2015, Akhter et al. reported an automated preparation scheme for Sn–Ag–Cu solder bumps, which is called the ball mounting method, successfully achieving the Flip-Chip bonding integration of a 90 × 90 pixel (80 µm pitch) Micro-LED array and a CMOS driver chip [86]. This research used the PacTech solder jetting system to precisely deposit Sn96.5%–Ag3%–Cu0.5% solder balls with a diameter of 50 µm on the gold-treated pads of the CMOS and completed the interconnection through reflow soldering in a nitrogen environment at 260 °C. The specific process is shown in Figure 20. Compared with the traditional bump-preparation process, this technology eliminates the patterned photolithography and stripping steps in the traditional process. The fully automated process (completing 8100 solder joints in 40 min) significantly improves the process efficiency. The test data shows that the interconnection structure presents a highly uniform solder joint morphology after a 30-s reflow. The driver chip successfully lights up the 4050-pixel Micro-LED array, with an initial yield of over 99%. However, as Micro-LEDs develop towards higher densities (for example, when the pixel size is reduced to 15 microns), the 50-micron bumps may be difficult to meet the requirements of future high-density interconnections due to size limitations.

### 4.3. Dip Soldering

**Figure 21:**
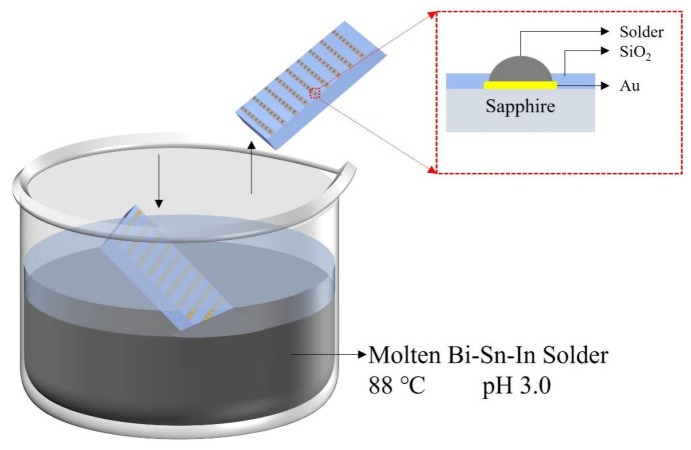
Dip soldering process.

In the field of Micro-LED device interconnection, the dip-soldering method is also an efficient option for bump fabrication. Its principle is based on the interaction between the molten solder and the substrate, where the surface tension is utilized to form the bump structure. Specifically, when the substrate is immersed in the molten solder, the solder will wet and spread on the surface of the metal pads. By precisely controlling the process of lifting the substrate, a uniform bump structure can be constructed with the help of surface tension. The advantage of the dip-soldering method is that it forms bumps by directly dipping into the molten solder, eliminating the need for the vacuum environment required in traditional evaporation or the complex electrochemical deposition process in electroplating [87]. However, the drawback is that the high-temperature molten solder may cause substrate warping or interfacial thermal stress. In 2023, Lee et al. ingeniously avoided the drawbacks of the dip-soldering method by using a low-melting-point solder of Bi32.5%–Sn16.5%–In51% (with a melting point of 60 °C) [88]. Moreover, they combined an acidic environment (pH 3.0) to inhibit the oxidation of the solder. The operation process is shown in Figure 21. The sapphire substrate was immersed in the molten solder and kept there for a period of time to allow the solder to fully wet the metal pads. Then, the substrate was slowly lifted, and hemispherical bumps with a height of 4 μm were successfully fabricated. Moreover, the research team proposed a parallel assembly technology based on Fluidic self-assembly (FSA). By adjusting the viscosity of the solution, they significantly improved the assembly efficiency and yield of small-sized chips (<100 μm). They completed the assembly of 19,000 GaN chips (with a diameter of 45 μm and a thickness of 5 μm) within 60 s, and the yield reached 99.88%, which was the highest level reported at that time.

### 4.4. Photosensitive Conductive Polymer Materials

**Figure 22:**
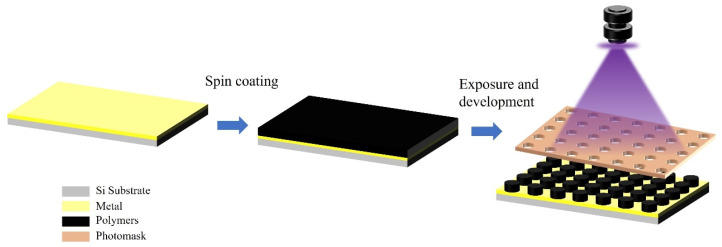
Carbon black bumps preparation process.

**Figure 23:**
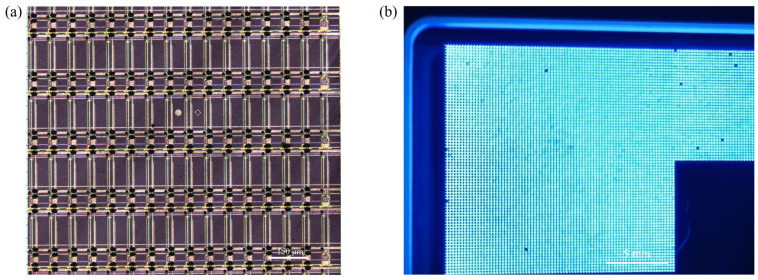
(**a**) 20× Magnified image of bumps under the optical microscope. (**b**) Illumination of an 80 × 80 Micro-LED array bonded to a TFT driver substrate.

Compared with traditional metal bump technologies, the photosensitive conductive polymer bump technology that has emerged in recent years demonstrates significant advantages in terms of material properties and fabrication processes. Photosensitive conductive polymers are fabricated by dispersing conductive carbon black in negative photoresist [89], and their electrical conductivity exhibits a characteristic S-shaped correlation with carbon black content [90,91]. When the carbon black content is below the critical threshold (percolation threshold), the composite material remains non-conductive. Upon exceeding this percolation threshold, the conductivity increases significantly with carbon black loading until reaching a saturation plateau [90,91,92]. Traditional metal bump-fabrication processes usually involve multi-step photolithography, metal evaporation, and complex stripping procedures. These processes are not only cumbersome but also use a large number of chemical solvents during production, which may pose potential risks to the environment and the health of operators. In 2024, Yang et al. used a photosensitive conductive polymer composite material containing carbon black nanoparticles as a substitute material for bumps and successfully fabricated carbon black bumps with a pitch of 8 μm on a gold-plated silicon wafer [93]. This material combines the characteristics of negative photoresist with inherent conductivity. It can be patterned through ultraviolet exposure to directly form microstructures with electrical interconnection functions. As shown in Figure 22, its fabrication process is simplified to: substrate pre-treatment → spin-coating to form a film → pattern exposure → development and curing, eliminating the metal deposition and stripping steps in traditional processes. In the same-year research, Zhu et al. further verified the process applicability of this photosensitive conductive polymer material on the TFT substrate [92]. As shown in Figure 23, the research team successfully fabricated a carbon-black composite bump array with a pixel pitch of 222 μm on the TFT driving backplane by optimizing the spin-coating parameters and exposure dose. Moreover, after bonding with the Micro-LED chips, the lighting effect was good. From the above research, it can be seen that the convenience and process compatibility of the new photosensitive conductive polymer material can provide a new technical path for the large-scale heterogeneous integration of Micro-LEDs.

### 4.5. Challenges and Limitations

This chapter evaluates four emerging bump-fabrication processes. Electroless plating, as a key advancement post-electroplating, eliminates uneven current density distribution [94] and bypasses conductive path construction (e.g., evaporation/sputtering) and post-removal steps (e.g., etching or DPSE [78]), simplifying the process. However, it requires precise control of metal salt, complexing agent, and reductant ratios for non-autocatalytic metals [95], resulting in instability and limited material options.

The advantages of ball mounting technology lie in its machine-controlled characteristics, offering superior process stability and operational efficiency compared to manual methods. By enabling precise bump placement through automated equipment, this technology eliminates conventional steps such as spin-coating photoresist, photolithography, and liftoff [86]. However, its limitations emerge as bump dimensions and pitches advance into the sub-10 μm regime: the physical constraint of nozzle aperture (bump diameter typically twice the nozzle size) restricts further miniaturization, while reported defects including poor solder wetting, solder depression, and localized ablation [96,97] degrade solder joint reliability. These challenges collectively hinder its application in high-end microelectronic packaging.

Dip soldering, as an operationally simplified joining technique, achieves bump formation through direct immersion of substrates into molten solder, effectively circumventing the requirement for specialized environments in conventional vacuum evaporation processes while streamlining the intricate electrochemical deposition procedures inherent in plating technologies [87]. Nevertheless, this process is predominantly constrained by its relatively low process yield, with welding quality being governed by multiple parametric interactions, including critical factors such as solder alloy composition, solder melting temperature, flux activation temperature, and immersion duration [98,99]. Furthermore, its capability for fabricating fine-pitch bumps remains to be thoroughly investigated. Optimization studies on dip soldering for patterned substrates reveal that distinct solder alloy systems necessitate specific immersion time windows to attain optimal bonding performance. Notably, repeated dipping operations not only fail to enhance the qualified product rate of patterned devices but may even substantially compromise process stability [87].

The fabrication of bumps using photosensitive conductive materials represents an innovative technology developed in recent years, which significantly reduces manufacturing cycles compared to conventional processes. This technique enables minimum bump spacing of 8 μm [93], demonstrating exceptional fine-pitch processing capability. Studies have shown that carbon black bumps fabricated on TFT driving substrates achieved 99.9% device yield when bonded with Micro-LED chips [92]. However, systematic investigations regarding mechanical stability and electrical/optical characteristics of these devices remain unreported, and their long-term reliability requires further validation.

## 5. Critical Analyses

Herein, we summarize the comparative advantages and limitations of six bump fabrication technologies:

1. Evaporation demonstrates notable merits in micron-level dimensional precision, highly dense film formation, and low defect density, making it extensively applicable for bump preparation. This technique is particularly suitable for multilayer heterogeneous material deposition through parameter optimization, fulfilling requirements for small-batch high-precision production with relatively low environmental impact. However, three critical limitations persist: restricted production efficiency, prohibitive equipment costs, and low material utilization rates.
2. Electroplating offers cost-effectiveness and batch-processing capabilities compared to evaporation. Nevertheless, non-uniform current density distribution induces height variations among bumps. Although Luo et al. and Tang et al. achieved bump-height uniformities of 2.83% and 2.26%, respectively, precise current density regulation remains a core technical challenge in large-scale commercial applications.
3. Electroless Plating eliminates current density heterogeneity inherent to electroplating while bypassing conductive layer preparation/post-processing steps. However, this method suffers from stringent chemical agent ratio control, limited material selectivity, low deposition rates, and environmental concerns associated with specific chemical reagents.
4. Ball Mounting exhibits advantages in automation accuracy and circumvention of conventional processes. Yet, it encounters miniaturization bottlenecks at sub-10-micron scales due to nozzle aperture constraints, coupled with reliability issues including poor solder wettability, dimple formation, and ablation defects.
5. Dip Soldering simplifies interconnection processes by directly immersing substrates into molten solder, eliminating specialized environments required for vacuum evaporation or electrochemical deposition. Nevertheless, this technology faces limitations in yield rates, sensitivity to multiple parameters (e.g., solder composition, melting temperature, flux activation temperature, immersion duration), and questionable capability for fine-pitch bump fabrication.
6. Photosensitive Conductive Polymer Materials have achieved recent breakthroughs in bump formation, featuring short manufacturing cycles and 8 μm ultra-fine pitch capabilities. This method attained 99.9% device yield in TFT driving substrate-to-Micro-LED bonding applications. However, systematic validation remains imperative for mechanical stability, long-term reliability, and optoelectronic performance characteristics.

Based on the comprehensive analysis of six bumping technologies, we propose that electroless plating exhibits the most promising development potential and may emerge as the dominant approach. Although material selectivity remains a limitation (particularly for depositing catalytically toxic metals such as indium [95]), contemporary Flip-Chip bonding primarily utilizes Cu, Sn, Au, and Ni, which all demonstrated to be achievable via optimized electroless plating processes, with current research also progressing in electroless in deposition [95,100]. Regarding high-density bump fabrication, electroless plating demonstrates comparable capability to that of electroplating and evaporation, though high-aspect-ratio structures necessitate the use of either electroless plating or electroplating. Lu et al. [34] and Tian et al. [25] have shown that when the bump diameter is around 3 μm, the height can be increased to 6 μm and 7.5 μm, respectively. Our experimental validation indicates that sub-1% bump uniformity surpasses alternative processes [84], with production throughput matching electroplating’s Tier-1 capacity, while cost-effectiveness benefits from eliminating power supply requirements. The EDS analysis by Tian et al. [25] verifies defect-free Ni bumps with dense microstructure, effectively mitigating performance degradation risks. Regarding process integration, it remains slightly inferior to evaporation due to the necessary considerations for substrate-chemical compatibility. From an environmental perspective, next-generation electroless systems exhibit markedly reduced ecological impact through the substitution of formaldehyde-based reducers [101] and cyanide-based complexing agents [81,82], aligning with green manufacturing trends. This research conclusion is premised on the condition that the long-term reliability of photosensitive conductive materials has not been sufficiently validated. Should subsequent experiments confirm the material’s reliable long-term stability, it will be necessary to re-evaluate and determine the optimal technical solution.

## 6. Conclusions and Outlook

In the field of Micro-LED device interconnection, we have systematically reviewed the technical challenges and optimization strategies of different bump-preparation methods. The research shows that although evaporation and electroplating are still the current mainstream interconnection technologies, electroless plating demonstrates significant advantages in terms of bump-height uniformity. Based on its unique process characteristics and scalability, electroless plating is expected to become the core technology for the next-generation high-precision bump preparation. However, its industrialization process still needs to overcome key challenges such as material compatibility and process efficiency. The thermomechanical properties of the mainstream materials used for bump fabrication in current Micro-LED applications are summarized in Table 3 (The thermomechanical properties summarized in Table 3 only include some key parameters of the mainstream materials for bump fabrication in current Micro-LED applications).

In the field of Micro-LED device interconnection, the selection of bump-preparation technology requires comprehensive consideration of precision, cost, efficiency, and environmental compatibility. Among traditional processes, the evaporation method still dominates high-resolution scenarios due to its advantages in high-precision thin-film deposition and uniformity. However, its high equipment cost and low material utilization rate restrict large-scale applications. The electroplating method is known for its controllable coating thickness and the ability to fill high-aspect-ratio structures, but it faces challenges in environmental pollution and substrate pre-treatment (if the substrate surface is not flat or clean enough, it may lead to uneven electroplating layers or defects, affecting device performance). Among emerging technologies, the electroless plating method performs well on non-conductive substrates and complex structures, but its slow plating speed and inferior coating performance compared to electroplating limit its popularization. The equipment for the ball-mounting method needs to be upgraded to meet the requirements of preparing high-density bumps (with a bump pitch of less than 10 μm). The dip-soldering method relies on low-melting-point alloys, but the cleaning of residues and the compatibility with heat-sensitive substrates remain bottlenecks. Although photosensitive conductive polymers streamline fabrication workflows, their long-term operational stability remains unvalidated. Future research should focus on the following directions:

1. Process integration: For example, combining processes such as evaporating the seed layer and electroplating bumps to balance precision and efficiency.
2. Green manufacturing: Developing environmentally friendly electroplating solutions and low-toxicity electroless plating reagents.
3. Material innovation: Exploring low-temperature bonding alloys and weather-resistant polymer composite materials.

As Micro-LEDs develop towards ultra-high-definition and flexibility, bump technology needs to make breakthroughs in micro-nano-scale interconnection, thermomechanical stability, and large-scale mass production to support the full commercialization of the next micro–structures generation display technology.

## Figures and Tables

**Table 1:** Comparison of bump-fabrication processes.

Process	Bump Size (μm)	Cost	Pitch (μm)	Process Complexity	Uniformity	Bump Materials
Evaporation	4.52	High	8	Low	High	Low-melting-point and easily evaporated metals
Electroplating	About 5	Medium	8	Medium	Medium	Most metals and alloys used in Flip-Chip
Electroless Plating	About 3	Low	8	Low	High	Ni, Cu, Au, Sn
Ball Mounting	50	Medium	80	Low	-	Low melting point alloy solder
Dip Soldering	About 40	Low	120	Medium	-	Low melting point alloy solder
PCPM	About 5	Medium	8	Low	High	Photosensitive conductive materials only

**Table 2:** Comparison of basic parameters of bumps.

Bump Material	Process	Bump Diameter	Bump Height	Bump Pitch	References
In	Evaporation	7.5 μm	About 3 μm	10.8 μm	[64]
In	Evaporation	10 μm	3 μm	15 μm	[65]
In	Evaporation	-	About 4.5 μm	8 μm	[66]
In	Evaporation	12 μm	2.7 μm	-	[67]
In	Evaporation	10 μm	4 μm	-	[68]
In	Evaporation	-	-	74 μm	[69]
Sn	Evaporation	-	3 μm	-	[70]
Sn	Evaporation	13.2 μm	-	15 μm	[71]

**Table 3:** Some thermomechanical properties of common bump materials [102].

Metal/Alloy	Melting Temp. (°C)	CTE (10^−6^ °C^−1^)	Conductivity (10^4^ Ω^−1^cm^−1^)	Yield Strength (MPa)
In	156.6	32.1	-	-
Sn	231.9	22	9.1	-
Cu	1083	16.5	58.8	56.4
Ag	960.5	18.9	62.1	-
Au	1063	14.3	45.5	308
Ni	1455	13.4	14.3	-
Sn96.5Ag3.5	221	22	9.0	39
Sn95.5Ag3.9Cu0.6	217	-	-	-

## Data Availability

No new data were created or analyzed in this study.
